# Layer-by-Layer Integration of Zirconium Metal–Organic Frameworks onto Activated Carbon Spheres and Fabrics with Model Nerve Agent Detoxification Properties

**DOI:** 10.1021/acsami.1c12095

**Published:** 2021-10-13

**Authors:** Rodrigo Gil-San-Millan, Pedro Delgado, Elena Lopez-Maya, Javier D. Martin-Romera, Elisa Barea, Jorge A. R. Navarro

**Affiliations:** Departamento de Química Inorgánica, Universidad de Granada, Av. Fuentenueva S/N, 18071 Granada, Spain

**Keywords:** composite, thin film, chemical warfare agent, filter, protective garments, heterogeneous catalysis

## Abstract

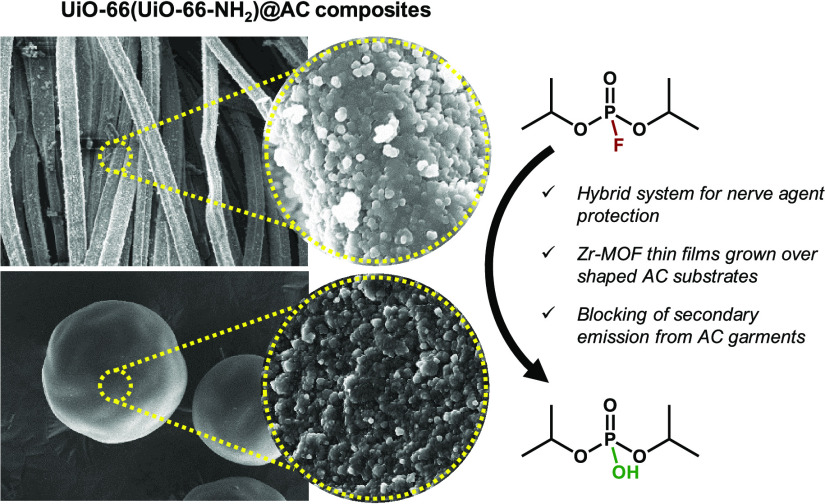

We report the controlled synthesis of thin films of prototypical zirconium metal–organic frameworks [Zr_6_O_4_(OH)_4_(benzene-1,4-dicarboxylate-2-X)_6_] (X = H, UiO-66 and X = NH_2_, UiO-66-NH_2_) over the external surface of shaped carbonized substrates (spheres and textile fabrics) using a layer-by-layer method. The resulting composite materials contain metal–organic framework (MOF) crystals homogeneously distributed over the external surface of the porous shaped bodies, which are able to capture an organophosphate nerve agent simulant (diisopropylfluorophosphate, DIFP) in competition with moisture (very fast) and hydrolyze the P–F bond (slow). This behavior confers the composite material self-cleaning properties, which are useful for blocking secondary emission problems of classical protective equipment based on activated carbon.

## Introduction

**Scheme 1:**
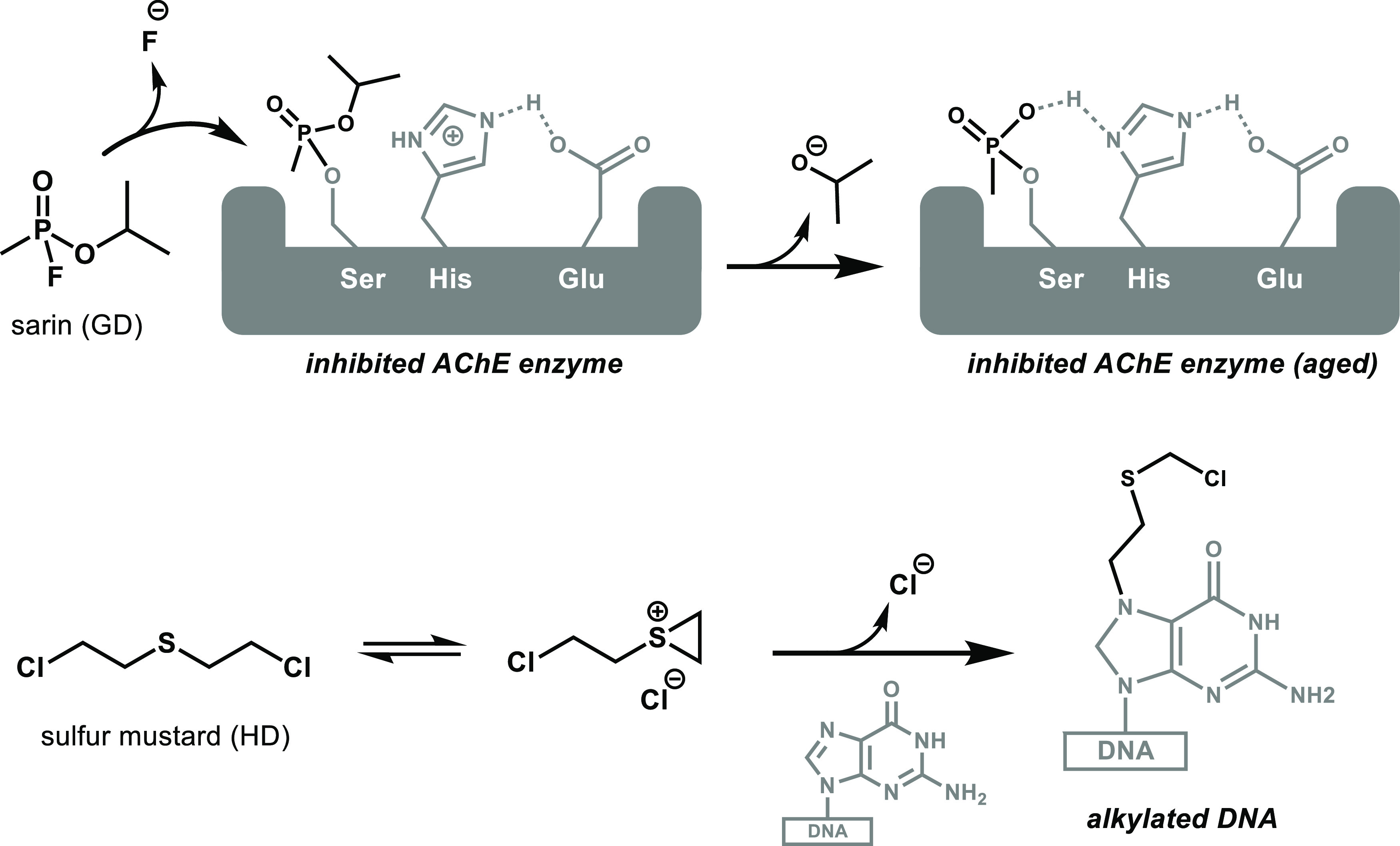
Toxicity Mechanism of Sarin (Nerve Agent) and Sulfur Mustard (Vesicant Agent)

Despite the international prohibition of the use and stockpiling of chemical warfare agents (CWAs), there is a high threat of exposure to highly toxic chemicals, as a consequence of destabilizing group actions. This problem has increased the demand for CWA detection and protective equipment.^1^ CWA toxicity is related to lipophilicity and the reactive nature of X–Y bonds (X = C, P; Y = F, Cl, N, S) typically found in the structure of these systems. These features lead to facile permeation through biological tissues and irreversibly binding to highly relevant biomolecules, namely, deoxyribonucleic acid (DNA) and acetylcholinesterase (AChE) for vesicant and nerve agents, respectively (Scheme 1).^2,3^ The ideal protective systems should be able to efficiently capture and decompose these extremely toxic chemicals before they come into contact with their biological target.

Current protective systems against CWAs take advantage of high porosity, hydrophobicity and easy shaping of activated carbon (AC) adsorbents.^4,5^ However, AC-based protective systems lack safety, as a small fraction of the adsorbed contaminant molecule can desorb from the pore surface (a phenomenon called secondary emission) and subsequently contaminate the user. In this regard, a variety of porous metal–organic frameworks (MOFs), constructed from metal clusters connected by multitopic organic ligands,^6^ behave as efficient catalysts for decontamination of nerve agents.^7−10^ However, as MOF materials are usually obtained in a powder form, they need to be shaped^11^ to allow their integration onto protective systems like suits, cartridge filters, or ventilation equipment. Recent efforts have been focused on the development of advanced protective materials based on catalytically active MOFs integrated onto textile fabrics^12−17^ combining the lightness and air permeation of the fibers. Still, it will be advantageous that the MOF support would have complementary adsorptive properties to increase the filter protection ability. In this regard, AC materials possess a broad range of pore sizes and are easily shaped in the form of spheres and textile fabrics suitable as filters. Nevertheless, MOF integration onto AC materials has been poorly explored.^18−21^

Herein, we report the controlled synthesis of two prototypical zirconium-based MOFs [Zr_6_O_4_(OH)_4_(benzene-1,4-dicarboxylate-2-X)_6_] (X = H, UiO-66; X = NH_2_, UiO-66-NH_2_) over the surface of activated carbon substrates in the form of fabrics (suited for the fabrication of protective suits) and spheres (suited as fillers on mask cartridges). We show that a layer-by-layer (LBL) synthesis is more advantageous than the *in situ* MOF synthesis with the LBL yielding systems containing Zr-MOF thin films homogeneously dispersed over the AC shaped bodies. Moreover, the resulting MOF@AC composites behave as self-cleaning materials in the capture and degradation of the organophosphate nerve agent simulant diisopropyl fluorophosphate (DIFP) blocking the secondary emission problems of traditional AC-based protective filters.

## Results and Discussion

**Scheme 2:**
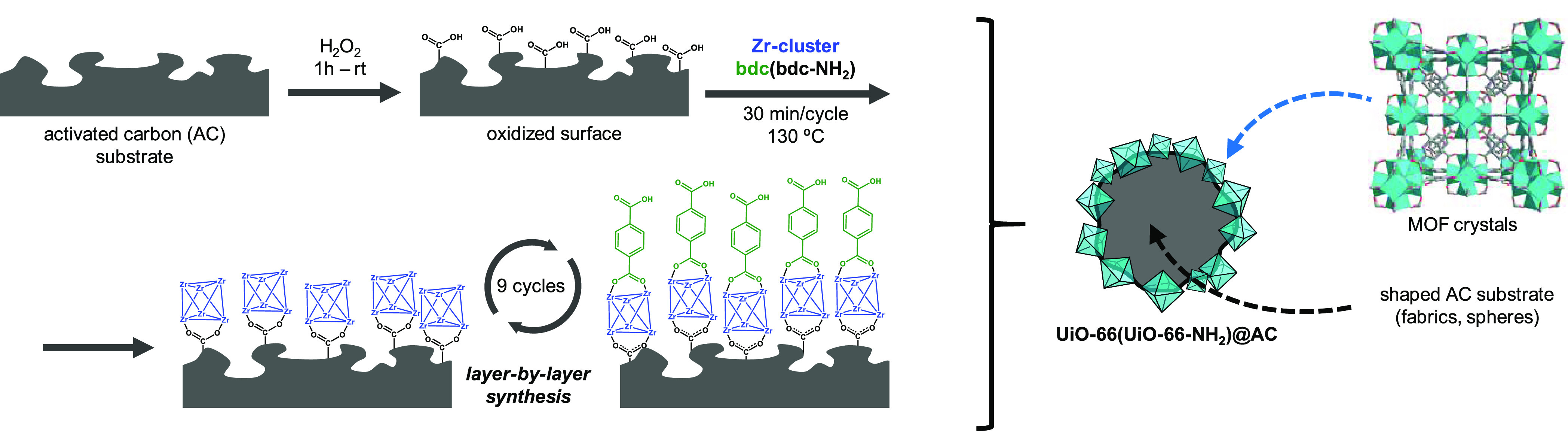
Synthesis of UiO-66(UiO-66-NH_2_)@AC (Fabrics, Spheres) Composites in a Layer-by-Layer Approach

Scheme 2 shows a general synthetic procedure for the layer-by-layer (LBL) MOF thin-film growth over the shaped carbonized substrates. In the first step, the carbonized substrate, in the form of fabrics and spheres, was treated with an oxidant to increase the concentration of oxygen groups (i.e., hydroxyl, carboxylate) on its surface to provide suitable metal-ion-anchoring sites. Temptative studies with nitric acid (HNO_3_) and hydrogen peroxide (H_2_O_2_) lead to the selection of the latter as it does not damage the AC porosity, as proven by N_2_ adsorption measurements (see below). This oxidative treatment led to an increase in oxygen groups on the surface as confirmed by X-ray photoelectron spectroscopy (XPS) analysis (see the Supporting Information, SI).

In the second step, two separate solutions containing the MOF precursors were prepared: (a) a preformed Zr_6_ oxocluster [Zr_6_O_4_(OH)_4_(AcO)_12_] solution in dimethylformamide (DMF)/AcOH^22^ and (b) a DMF solution of the organic spacer (4-benzenedicarboxylic acid (H_2_bdc) or amino-1,4-benzenedicarboxylic acid (H_2_bdc-NH_2_)) (see the SI). The LBL crystallization process consisted of the alternate immersion of the AC substrate in the zirconium cluster and linker solutions (Scheme 2). A washing step with DMF was carried out between transfers of the AC material into the different solutions to remove any unreacted MOF precursor, thus avoiding MOF crystallization outside of the carbon shaped bodies. In this manner, strongly attached Zr-MOF films were constructed for all materials. The resulting composites were labeled **UiO-66@AC(fabrics, spheres)** and **UiO-66-NH**_**2**_**@AC(fabrics, spheres)**.

**Figure 1:**
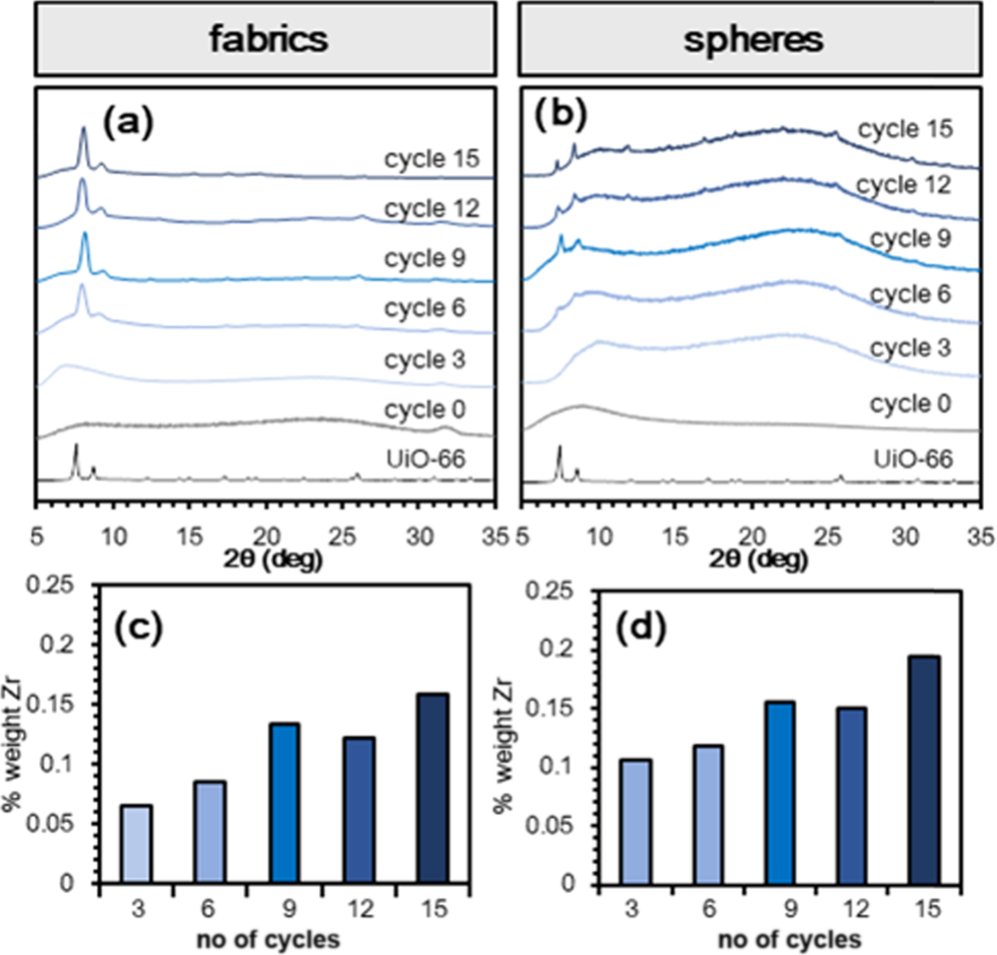
Study of UiO-66 growth over shaped activated carbon (AC) substrates. PXRD patterns for UiO-66@AC composite materials after different number of cycles for (a) fabrics and (b) spheres. Zr weight percentage after different number of cycles for (c) fabrics and (d) spheres, as determined by ICP-MS.

The MOF thin-film growth process was carried out using 1 h immersion at 130 °C in each component solution. A combined study of powder X-ray diffraction (PXRD), inductively coupled plasma-mass spectrometry (ICP-MS) (Figure 1a–d), and thermogravimetric analysis (TGA) (Figures S7 and S8) over 15 growth cycles shows the formation of a crystalline UiO-66 phase on the surface of AC materials after approximately three cycles. The MOF content in the materials increases with growth cycles. Similarly, Fourier transform infrared spectroscopy (FTIR) results (Figure S6) showed that vibrational bands associated with the bdc ligand (1100–1800 cm^–1^ region) increase with the increasing number of growth cycles. The amount of deposited UiO-66 thin films, after nine cycles, was estimated to reach 0.422 and 0.496 wt % MOF content for AC fabrics and spheres, respectively.

**Figure 2:**
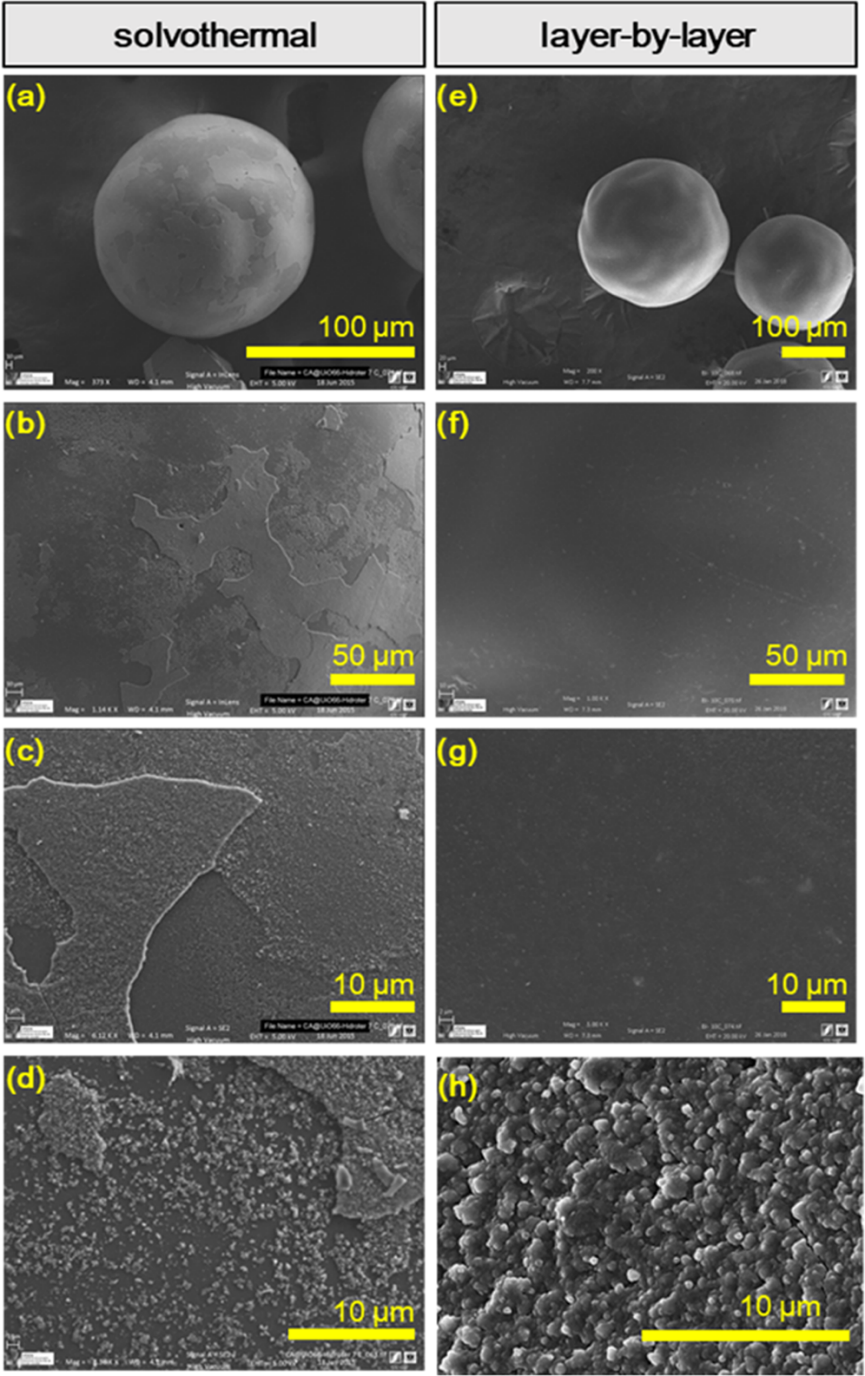
Comparison between solvothermal and LBL syntheses. SEM images at three different magnifications for (a–d) solvothermally prepared and (e–h) LBL-prepared (cycle 10) composites (2 h cycles at 130 °C). For comparison, Figure S9 shows SEM images for pristine fabrics and spheres.

**Figure 3:**
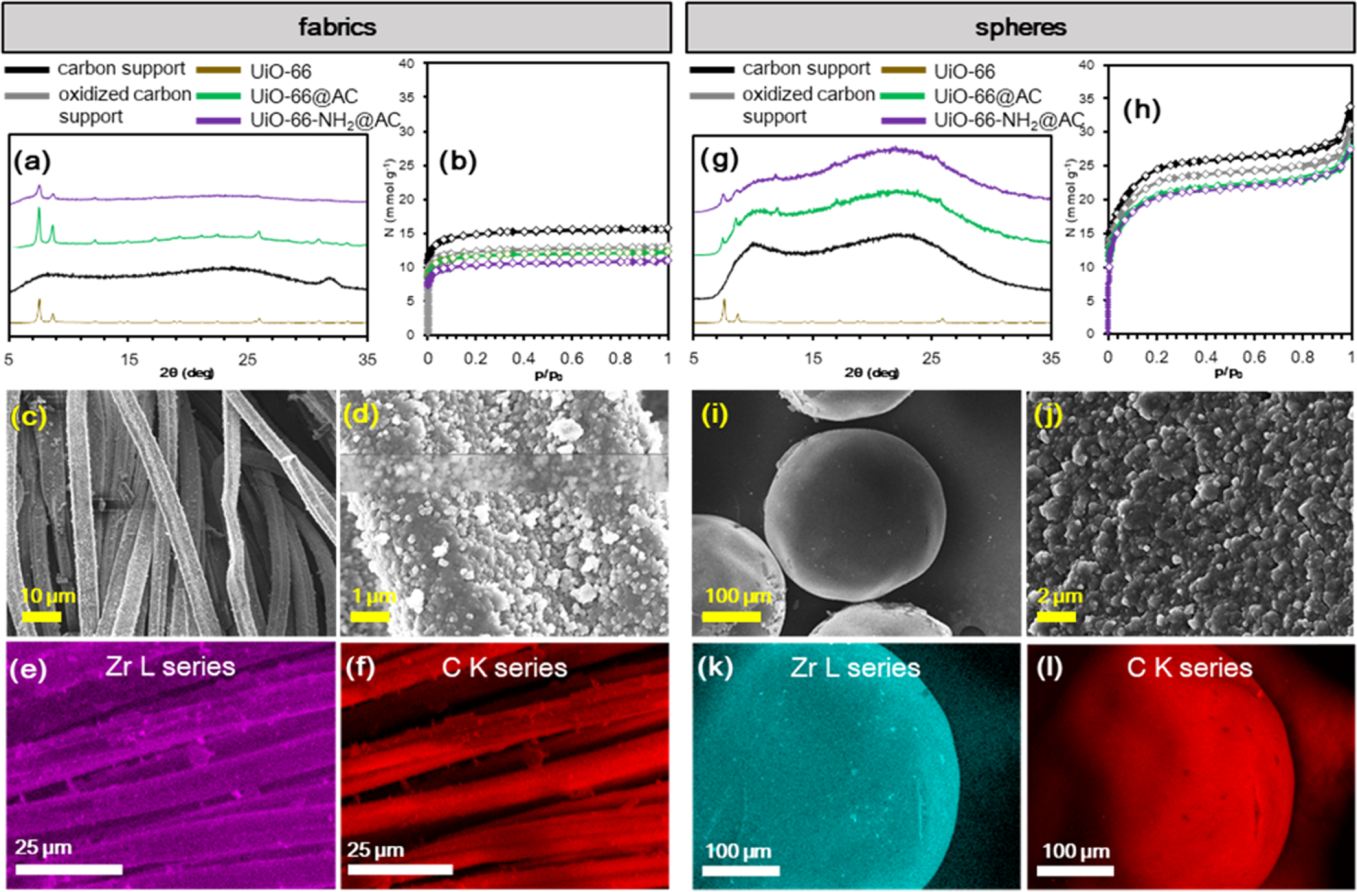
Characterization of Zr-MOF@AC composites (nine cycles, 130 °C, 30 min each cycle). PXRD patterns for (a) fabrics and (g) spheres and nitrogen adsorption isotherms for (b) fabrics and (h) spheres. SEM–energy dispersive X-ray analysis (EDX) study of (c–f) **UiO-66@AC(fabrics)** and (i–l) **UiO-66@AC(spheres)**. Color code: pristine carbon (black), H_2_O_2_-mediated oxidized carbon (gray), UiO-66@CA (green), and UiO-66-NH_2_@CA (purple).

In a parallel study, we also addressed the UiO-66 typical solvothermal synthesis in the presence of shaped carbon materials. With this aim, the MOF precursors were mixed in a solvothermal reactor and heated at an elevated temperature (220 °C) and pressure overnight. This process was repeated during nine growth cycles leading to the formation of microcrystalline UiO-66 both attached to the shaped AC together with a large excess of loose MOF materials. Scanning electron microscopy (SEM) images show a heterogeneous distribution of UiO-66 microcrystals on the external surface of the AC spheres and fibers giving rise to terraces and granules (Figure 2a–c). The observed behavior for the solvothermal-prepared UiO-66@AC composites is in clear contrast to a homogeneous film formation observed for the LBL-prepared composites (Figures 2e−h and 3).

In a second stage in this work, we prepared a new set of MOF@AC composite materials of UiO-66 and UiO-66-NH_2_ networks, reducing the time length of each growth cycle from 2 h to 30 min over nine cycles. The results show the successful preparation of **UiO-66@AC(fabrics, spheres)** and **UiO-66-NH**_**2**_**@AC(fabrics, spheres)** composites. PXRD patterns (Figure 3a,g) show the presence of crystalline MOF over the surface of all composites. TGA analysis (Figures S7 and S8) shows an increased pyrolytic residue for the MOF@AC composite materials indicative of the presence of ZrO_2_ from the Zr-MOF layer. ICP analysis (Table 1) showed Zr wt % values ranging from 0.022 to 0.051 corresponding to 0.07–0.16% MOF incorporation on the MOF@AC composites (Table 1). SEM–EDX mapping analyses (Figures 3c–f,i–l and S10) show a homogeneous dispersion of Zr and MOF crystals on the external surface of the activated carbon shaped bodies. A cross-sectional elemental analysis of the fabric fiber showed a Zr signal only on the outer surface of the fibers with a layer thickness of approximately 300 nm (Figure S10). This is indicative of the preferential formation of the MOF thin-film layer at the hydrophilic external surface (oxygen groups) of AC shaped bodies with no MOF formation onto the inner hydrophobic AC pore surface. Nitrogen adsorption isotherms indicated that both the MOF and AC porous structures are accessible in the MOF@AC composites (Figure 3b,h and Table 1). A drop in the adsorption capacity of the activated carbon material of 19% for AC fabric and 9% for AC spheres is noticed upon the oxidation treatment, which can be related to the partial degradation of materials. A further adsorption capacity drop is also noticed after the MOF layer deposition of around 5 and 15% for the fabric and spheres, respectively, which might be the related to hindered diffusion of N_2_ molecules through the microporous MOF layer and/or adsorbed organic linkers, thereby reducing their associated porosity. Slightly lower adsorption capacities for UiO-66-NH_2_@AC composites are attributed to the steric effects of the NH_2_ group.

**Table 1:** Textural and Compositional Characteristics of MOF@AC Materials

material	*V*_p_ (cm^3^ g^–1^)	*S*_BET_ (m^2^ g^–1^)	Zr wt %	estimated MOF wt %	estimated MOF in the catalyst (mg)
carbon fabrics	0.50	1070			
oxidized carbon fabrics	0.42	1040			
UiO-66@AC(fabrics)	0.40	840	0.051	0.16	0.06
UiO-66-NH_2_@AC(fabrics)	0.34	745	0.048	0.15	0.06
carbon spheres	0.87	2045			
oxidized carbon spheres	0.80	1880			
UiO-66@AC(spheres)	0.73	1520	0.041	0.12	
UiO-66-NH_2_@AC(spheres)	0.72	1495	0.022	0.07	

**Figure 4:**
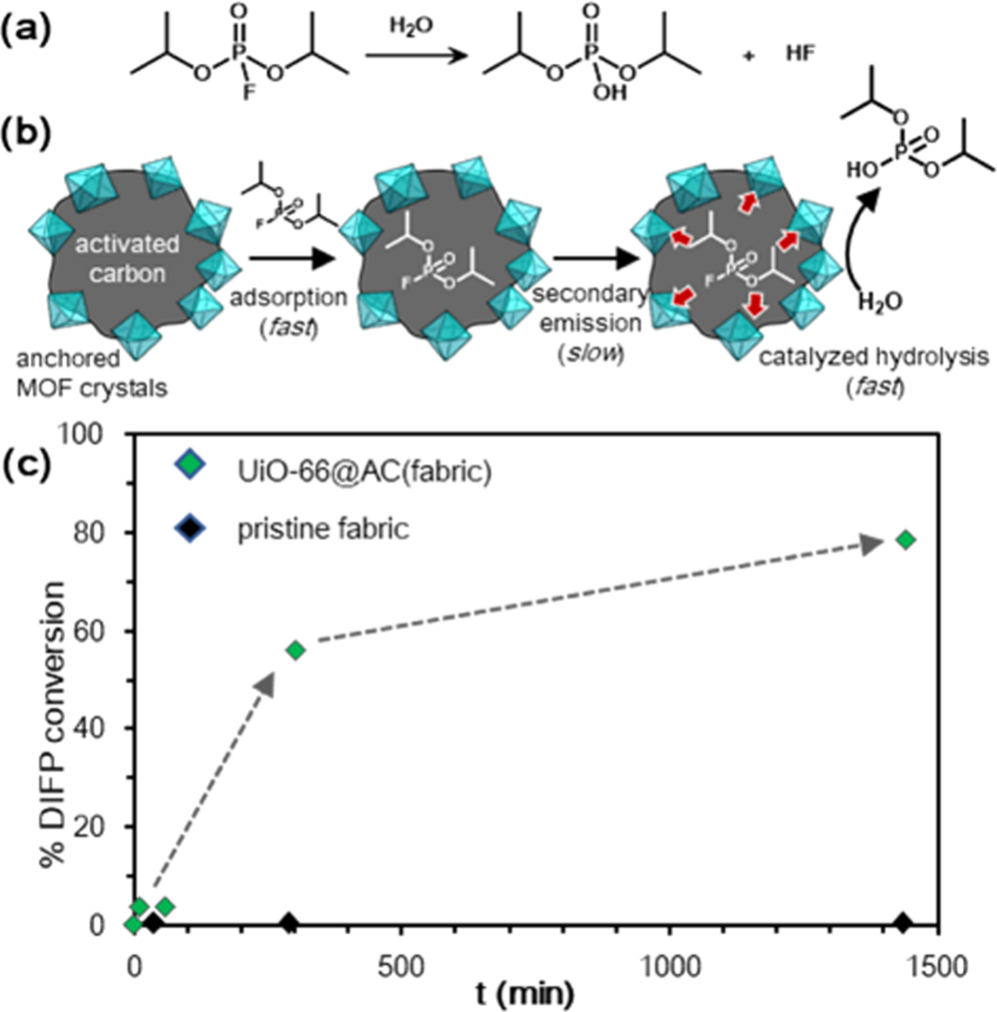
Blocking of the secondary emission by MOF@AC materials. (a) DIFP hydrolytic reaction. (b) Proposed three-step mechanism for the synergistic detoxification of DIFP on the composite surface. (c) DIFP hydrolysis kinetic study. Reaction conditions: 40 mg of the MOF@AC catalyst, 1.25 μL of DIFP, 10 μL of H_2_O, MOF/DIFP ratio of approximately 1:5, 60 °C. Extraction with CH_2_Cl_2_ (0.5 mL) at 1, 10 min, 1, 5, and 24 h.

Once we structurally characterized the MOF@AC composites, we proceeded to evaluate their possible functionality for detoxification of CWAs, as a proof of concept for self-cleaning materials. With this aim, we have evaluated the activity of **UiO-66(UiO-66-NH**_**2**_**)@AC(fabrics)** in the catalytic hydrolysis of the nerve agent simulant diisopropyl fluorophosphate (DIFP) (Figure 4a). For this reaction, 40 mg of the MOF@AC catalyst was impregnated with 10 μL of H_2_O and 1.25 μL of DIFP (MOF/DIFP ratio of approximately 1:5) in a closed vial. Subsequently, the vials were incubated at 60 °C for increasing time periods at 1, 10 min, 1, 5, and 24 h, followed by the extraction with CH_2_Cl_2_ (0.5 mL) and analysis by GC. The results are indicative of self-cleaning behavior for **UiO-66@AC(fabric)** and **UiO-66-NH**_**2**_**@AC(fabric)** composites with respective 80 and 40% DIFP detoxification after 24 h (Figures 4c and S7). Figure 4b shows the plausible mechanism of the three-step model nerve agent detoxification process. In the first step, fast and strong adsorption of DIFP on the hydrophobic activated carbon pore structure takes place, accounting for the traditional protective behavior of activated carbon materials. In the second step, a slow equilibrium takes place between the physisorbed contaminant (secondary emission) and the MOF particles that are distributed over the external surface of the activated carbon fibers. Finally, DIFP is hydrolyzed on the Zr-MOF pore structure, as shown in previous works.^23−25^ The observed lower performance of **UiO-66-NH**_**2**_**@AC(fabric)** might be related to a pH effect of unbuffered media as recently found by Cohen and co-workers.^26^ We assume the secondary emission diffusion process as the rate-limiting step in this detoxification process. In this regard, DIFP secondary emission is only appreciable above room temperature, as demonstrated previously by our group for AC Blücher spheres.^27^ It should be noted that the pristine AC fabrics do not give rise to any DIFP degradation under similar conditions, which indicated the benefit of the incorporation of the MOF layer on the AC fabrics to yield self-cleaning adsorbent materials.

Nevertheless, a similar study on the UiO-66@AC(spheres) composites is indicative of no improvement in the detoxification properties upon incorporation of the MOF layer (Figure S13), which can be attributed to the strong competition of the adsorption process on the spheres pore structure with the detoxification properties on the UiO-66 layer. This result correlates with the much higher adsorption capacity and Brunauer–Emmett–Teller (BET) surface of AC spheres in comparison to AC fabrics.

## Conclusions

The controlled layer-by-layer growth of crystalline MOF thin films over AC shaped substrates can be regarded as a suitable approach for the fabrication of advanced self-cleaning porous materials. The characterization results demonstrated the high dispersion of Zr-MOF crystals, and catalysis results showed their efficiency in the blocking of the secondary emission problem of classical CWA protective garments. We can conclude that the combination of two porous materials of different characteristics (porous hydrophobic carbon and catalytically active hydrophilic MOF) is a suitable way of improving the features of activated carbon traditional protective filters. Further work needs to be carried out to find a suitable balance between physisorption of CWA on a hydrophobic adsorbent and hydrolytic detoxification on a hydrophilic MOF to bring these composites to real applications.

## Supporting Information Available

Details of experimental procedures, composite preparation, and characterization (PDF)
