# Supplementary material for: Layer-by-Layer Integration of Zirconium Metal–Organic Frameworks onto Activated Carbon Spheres and Fabrics with Model Nerve Agent Detoxification Properties
Source: ACS Appl Mater Interfaces. 2021 Oct 13;13(42):50491–6. doi: 10.1021/acsami.1c12095 (PMC8554759; doi:10.1021/acsami.1c12095)
Supplement: Supplementary file 1 — am1c12095_si_001.pdf [file am1c12095_si_001.pdf]

SUPPORTING INFORMATION FOR:

Layer-By-Layer Integration of Zirconium-Metal-Organic Frameworks onto Activated Carbon Spheres and Fabrics with Model Nerve Agent Detoxification Properties

Rodrigo Gil-San-Millan[†,‡], *Pedro Delgado*[†], *Elena Lopez-Maya*[†,] , *Javier Romera*[†], *Elisa Barea*[†] *and Jorge A. R. Navarro*[*,†]

† Departamento de Química Inorgánica, Universidad de Granada, Av. Fuentenueva S/N, 18071, Granada, Spain.

*e-mail: jarn@ugr.es

‡ Faculty of Chemistry, University of Wrocław, F. Joliot-Curie 14, 50-383 Wrocław, Poland.

Chemistry Department & CICECO, University of Aveiro, 3810-193 Aveiro, Portugal

Table of contents

S1. General considerations: starting materials and characterization S-3

S1.1. Materials and reagents **S-3**

S1.2. Physical and chemical characterization **S-3**

S2. Synthesis of the materials S-4

S3. Chemical characterization S-6

S3.1. X-Ray photoelectron spectroscopy (XPS) **S-6**

S3.2. Fourier Transform Infrared Spectroscopy (FTIR) **S-11**

S3.3. Thermogravimetrical Analysis (TGA) **S-12**

S3.4. Scanning Electron Microscopy - Energy Dispersive X-ray spectroscopy (SEM-EDX) **S-14**

S4. Catalytic tests S-17

S5. References S-19

S1. General considerations: starting materials and characterization

S1.1. Materials and reagents

Chemical reagents and solvents were purchased at commercial sources and used without additional purification. Carbonaceous fabrics were obtained from Zorflex®, while carbon spheres were obtained from Blücher®.

S1.2. Physical and chemical characterization

X-Ray powder diffraction (XRPD) data were obtained on a D2 PHASER Bruker diffractometer using Cu Kα radiation (λ= 1.5418 Å) by means of a scan in the 5-35° 2θ range with 0.05° steps. Carbonaceous substrates were deposited in the hollow of a zero-background silicon sample holder.

-Nitrogen adsorption isotherms were measured at 77 K on Micromeritics Tristar 3000 and 3flex volumetric instruments. Prior to measurement, powder samples were heated at 423 K for 7 h and outgassed to 10^{-1} Pa.

-Thermogravimetric Analysis (TGA) was carried out by a METTLER-TOLEDO mod. TGA/DSC1 system.

-Scanning electron microscopy (SEM) images and element mapping (EDX) were obtained on a Carl Zeiss SMT AURIGA (FIB-FESEM) system.

-^{1}H NMR spectra were recorded on a 400 MHz BRUKER Nanobay Avance III HD spectrometer.

-FTIR spectra were recorded on a Bruker Tensor 27 IR equipped with a PIKE GladiATR ATR module

-ICP-MS measurements for zirconium quantification were obtained on a NexION 300D with plasma torch ionization source and quadrupole ion filter.

S2. Synthesis of the materials

- *Zirconium oxohydroxide cluster solution*: $Zr_6O_6(OH)_6(AcO)_{12}$ cluster solution was prepared according to the procedure reported by Farha et al.[1] 355 µL of a $Zr(OPr)_4$/PrOH solution were mixed with 35 mL of DMF and 20 mL of acetic acid in a 100 mL round bottom flask. The mixture was heated at 130°C without stirring for two hours, when a yellow-orange solution appeared.

- *Ligand solution*: 1,4-benzenedicarboxylic acid (**H_2bdc)** solution (**UiO-66@AC** synthesis) was prepared by dissolving 375 mg (2.25 mmol) of **H_2bdc** in 50 mL of DMF. Similarly, 2-amino-1,4-benzenedicarboxylic acid (**H_2bdc-NH_2**) solution (**UiO-66-NH_2@AC**) was prepared by dissolving 408.9 mg (2.25 mmol) of **H_2bdc-NH_2** in 50 mL of DMF.

- *Layer-by-layer synthesis of* ***Zr-MOF@AC(fabrics, spheres)*** *composites*: On a first step, 10 mL of H_2O_2 (30% v/v) were added to 200 mg of activated carbon (Blücher® spheres or Zorflex® fabrics), and the oxidation reaction was left standing at room temperature for 1 hour (a bubbling is observed). The resulting oxidized carbon was cleaned 3 times with water, and dried on an oven at 70°C.

On a second step, the oxidized carbon was suspended in the *cluster solution* at 130°C for 15 min, washed three times with DMF, and then added to the *ligand solution* at 130°C for another 15 min. Again, the sample was washed three times with DMF and added back to the *cluster solution* completing then a 30-min-cycle. This procedure was repeated successively

making several cycles. Finally, the sample was washed 3 times with THF, suspended in THF overnight, and dried at 70°C.

- *Solvothermal synthesis of* ***Zr-MOF@AC(spheres)-ht*** *composite*: The material was prepared as reported elsewhere[2] but including the carbonaceous substrate. In this manner, 100 mg of shaped activated carbon material was added to a mixture of 1.29 g $ZrCl_4$, 1.84 g terephthalic acid, 0.925 mL HCl and 37.5 mL DMF and transferred to a teflon-lined hydrothermal flask. The flask was then placed in an oven at 220° C for 16h. Afterwards, the physical mixture of MOF@AC composite and the exces of unsupported MOF were separated. The composite was then washed with DMF for three times to remove both unreacted precursors and suspended crystalline MOF particles. This procedure was repeated successively making up to 9 cycles.

S3. Chemical characterization

S3.1. X-Ray photoelectron spectroscopy (XPS)

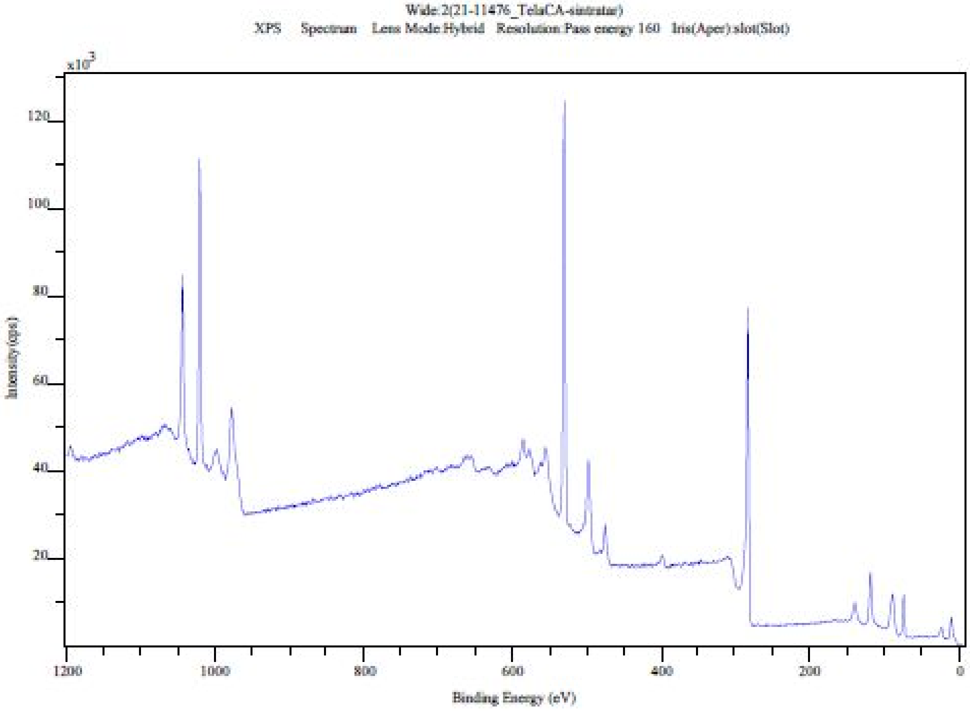

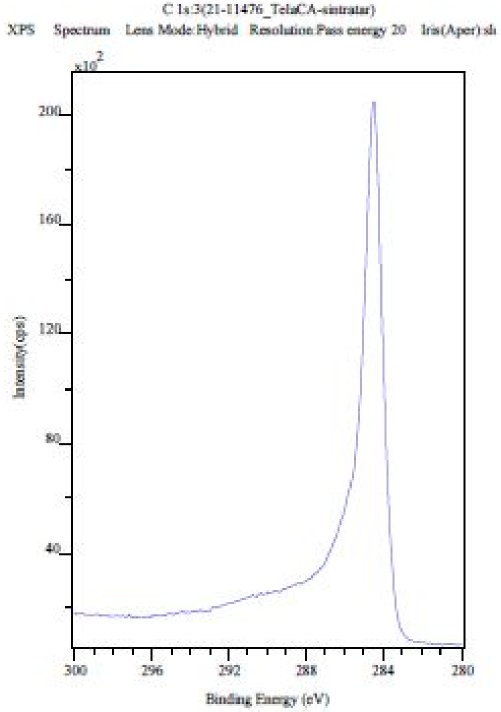

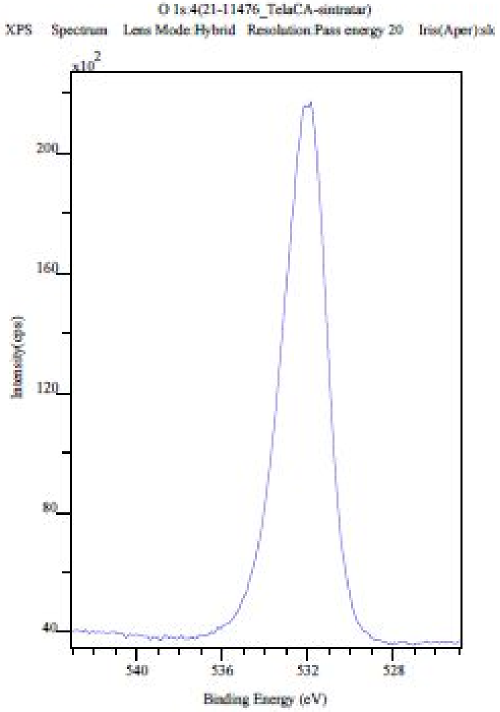

Figure S1. XPS spectra for pristine activated carbon fabric.

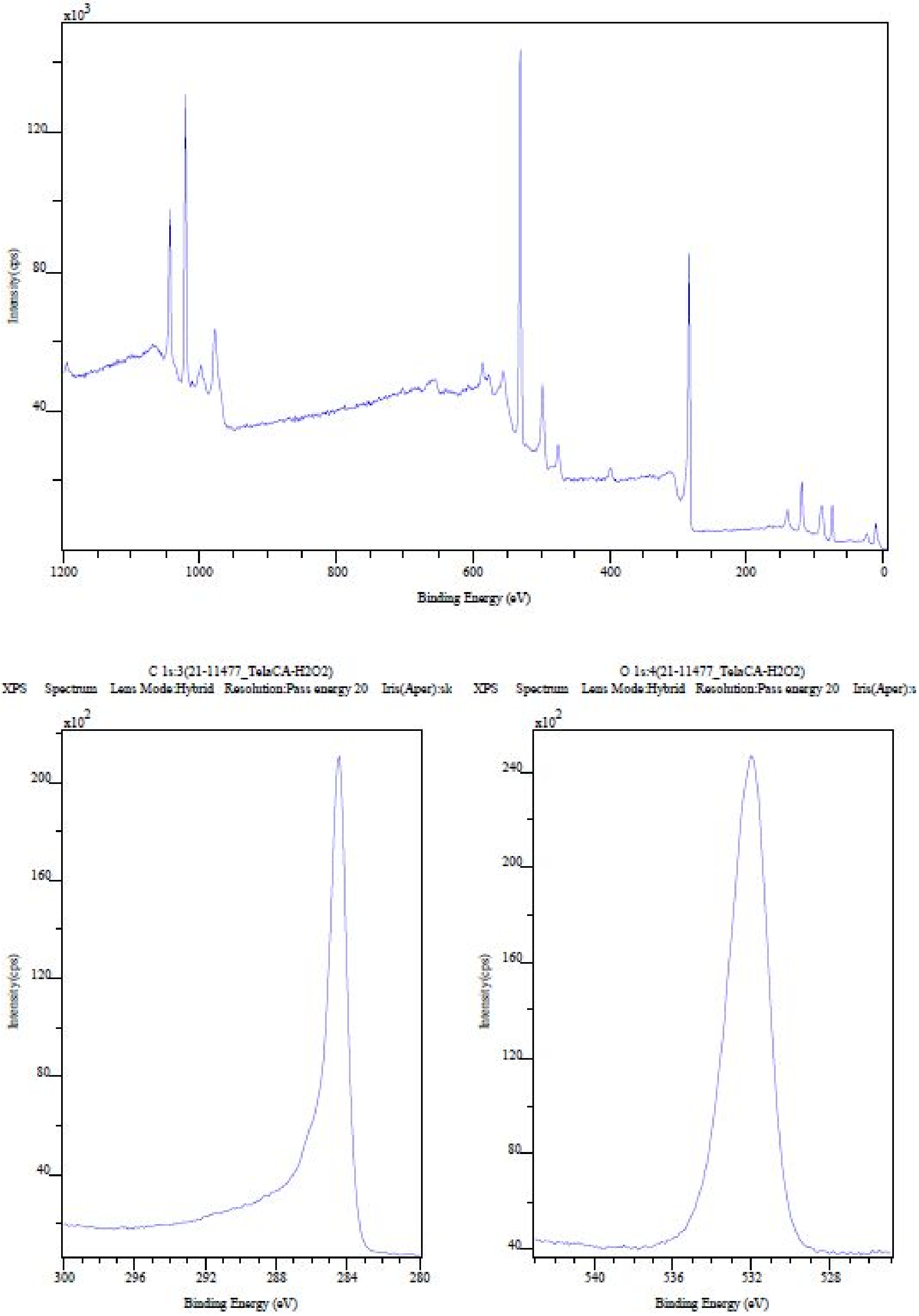

Figure S2. XPS spectra for activated carbon fabric after H_2O_2 treatment.

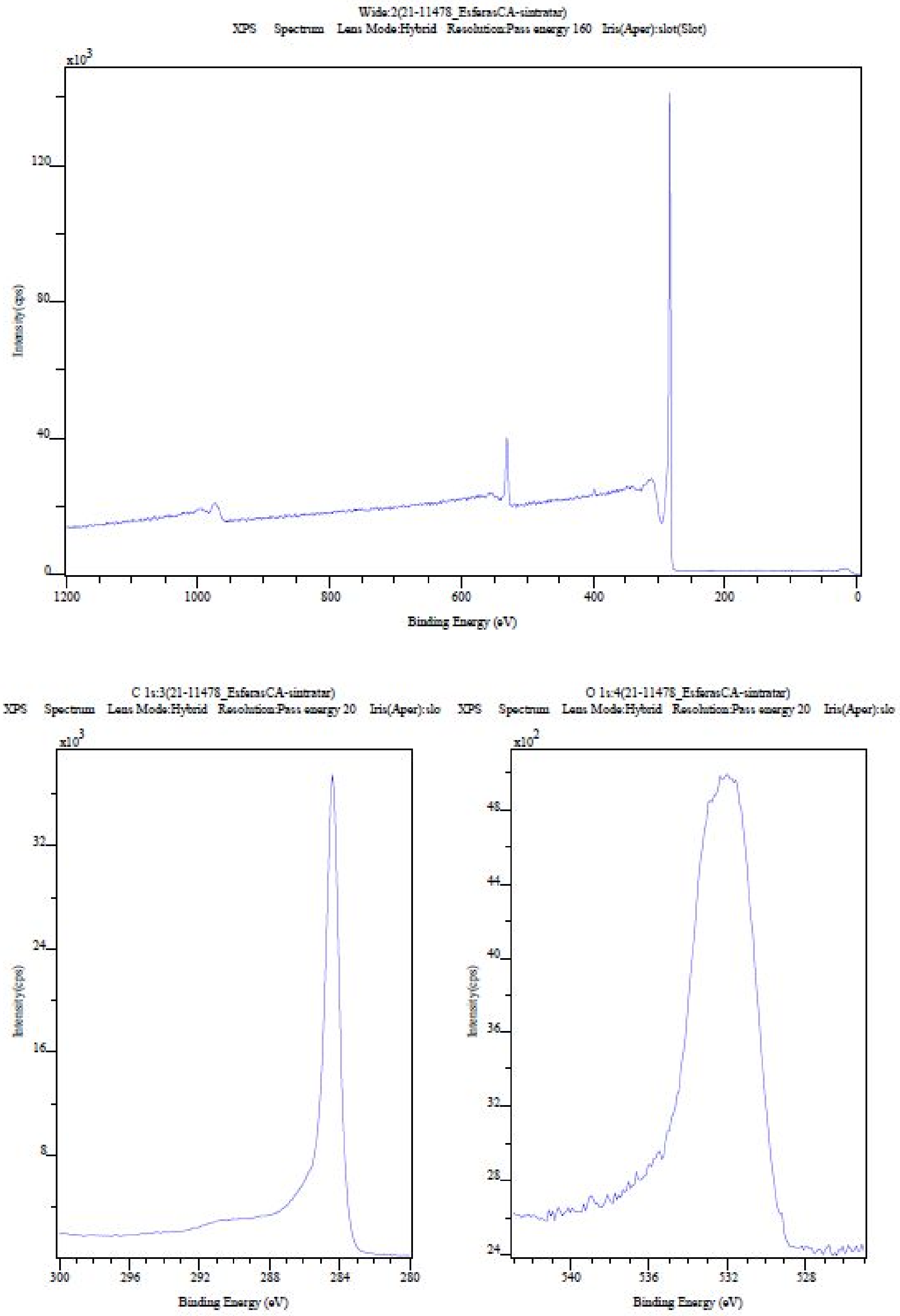

Figure S3. XPS spectra for pristine activated carbon spheres.

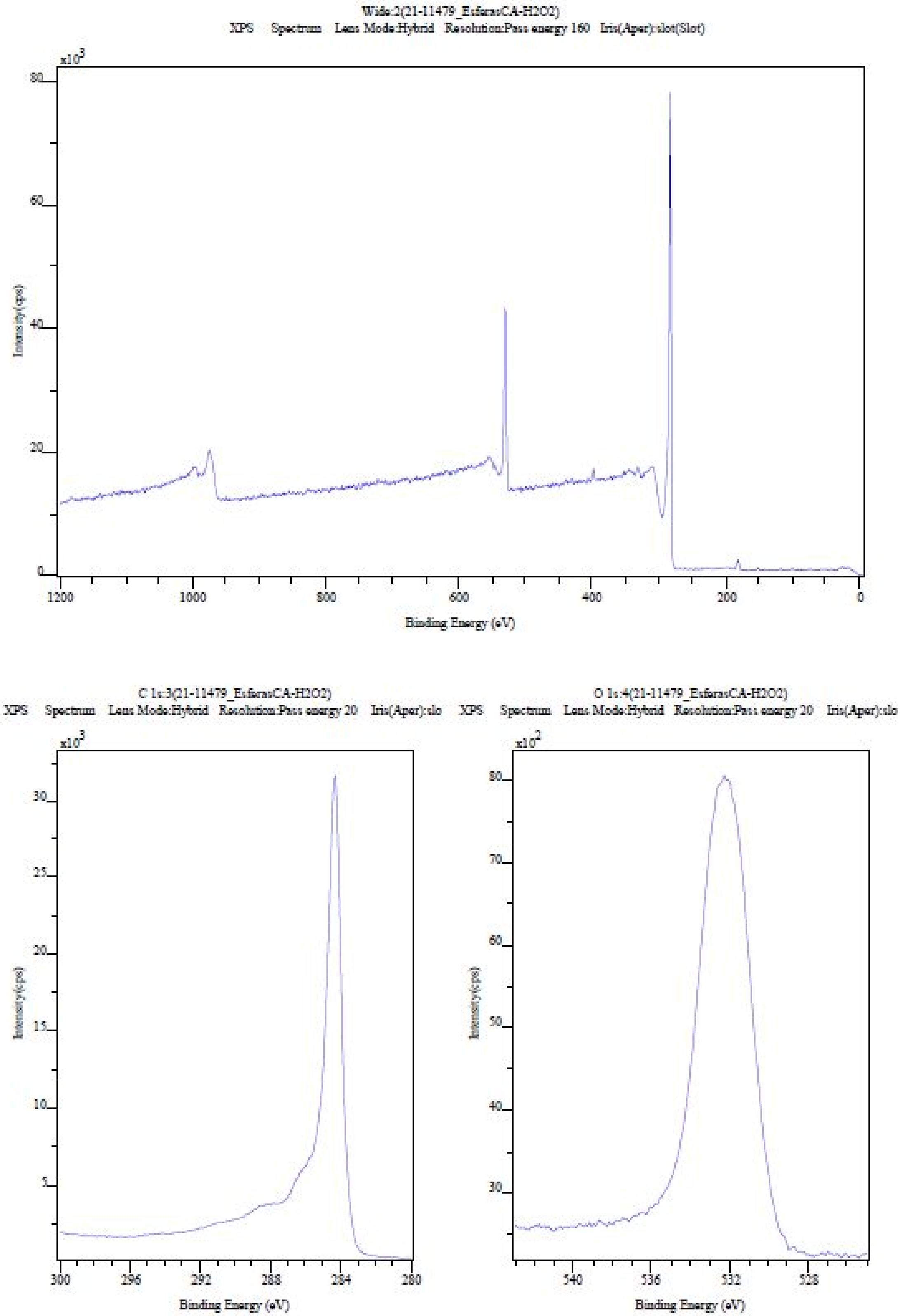

Figure S4. XPS spectra for activated carbon spheres fabric after H_2O_2 treatment.

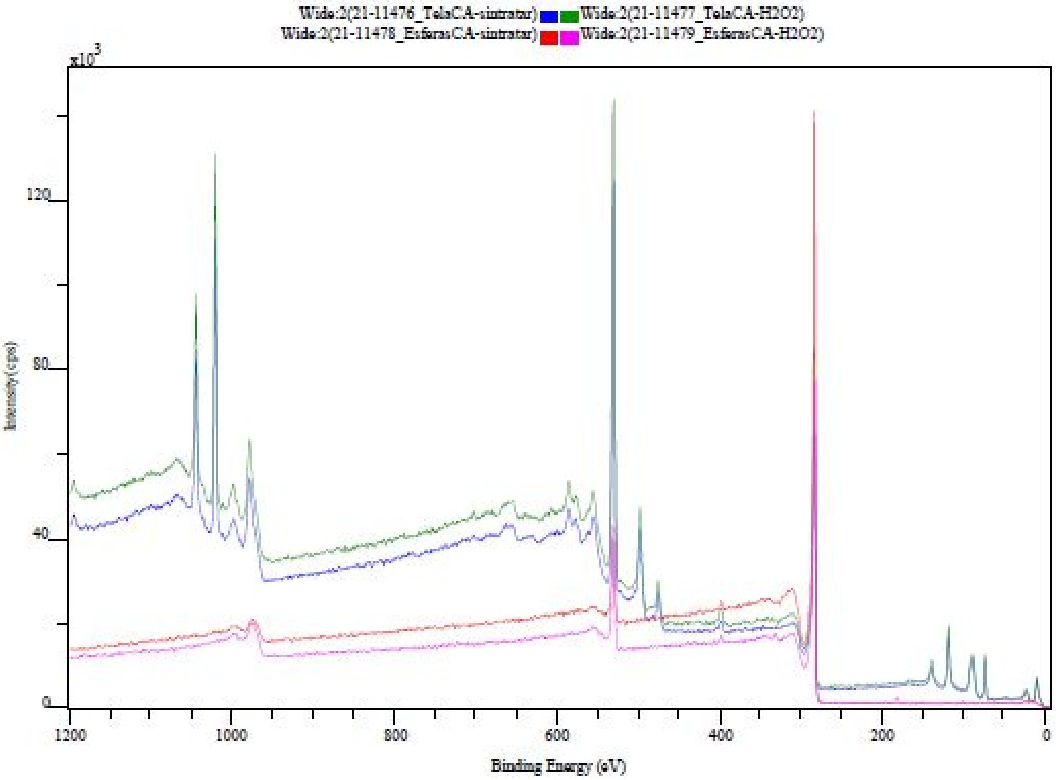
Wide:2(21-11476_TelaCA-sintratar)
Wide:2(21-11477_TelaCA-H2O2)
Wide:2(21-11478_EsferasCA-sintratar)
Wide:2(21-11479_EsferasCA-H2O2)
x10^3
Intensity (cps)
Binding Energy (eV)
0
40
80
120
1200
1000
800
600
400
200
0

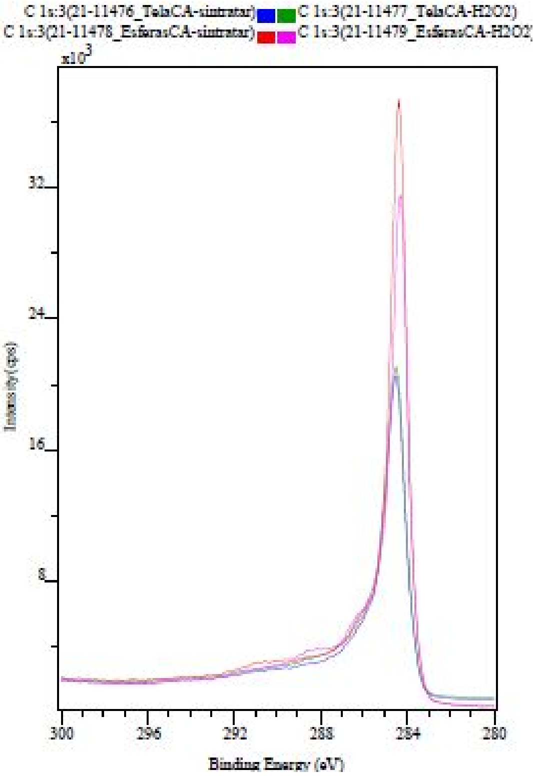
C 1s:3(21-11476_TelaCA-sintratar)
C 1s:3(21-11477_TelaCA-H2O2)
C 1s:3(21-11478_EsferasCA-sintratar)
C 1s:3(21-11479_EsferasCA-H2O2
x10^3
Intensity (cps)
Binding Energy (eV)
8
16
24
32
300
296
292
288
284
280

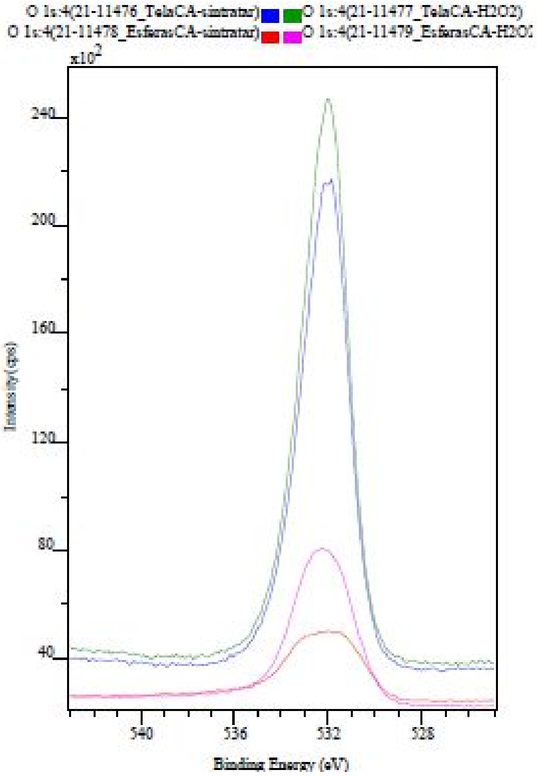
O 1s:4(21-11476_TelaCA-sintratar)
O 1s:4(21-11477_TelaCA-H2O2)
O 1s:4(21-11478_EsferasCA-sintratar)
O 1s:4(21-11479_EsferasCA-H2O
x10^2
Intensity (cps)
Binding Energy (eV)
40
80
120
160
200
240
540
536
532
528

Figure S5. Comparative XPS spectra and elemental quantification for carbonaceous substrates before and after H_2O_2 oxidation.

S3.2. Fourier transform infrared spectroscopy (FTIR)

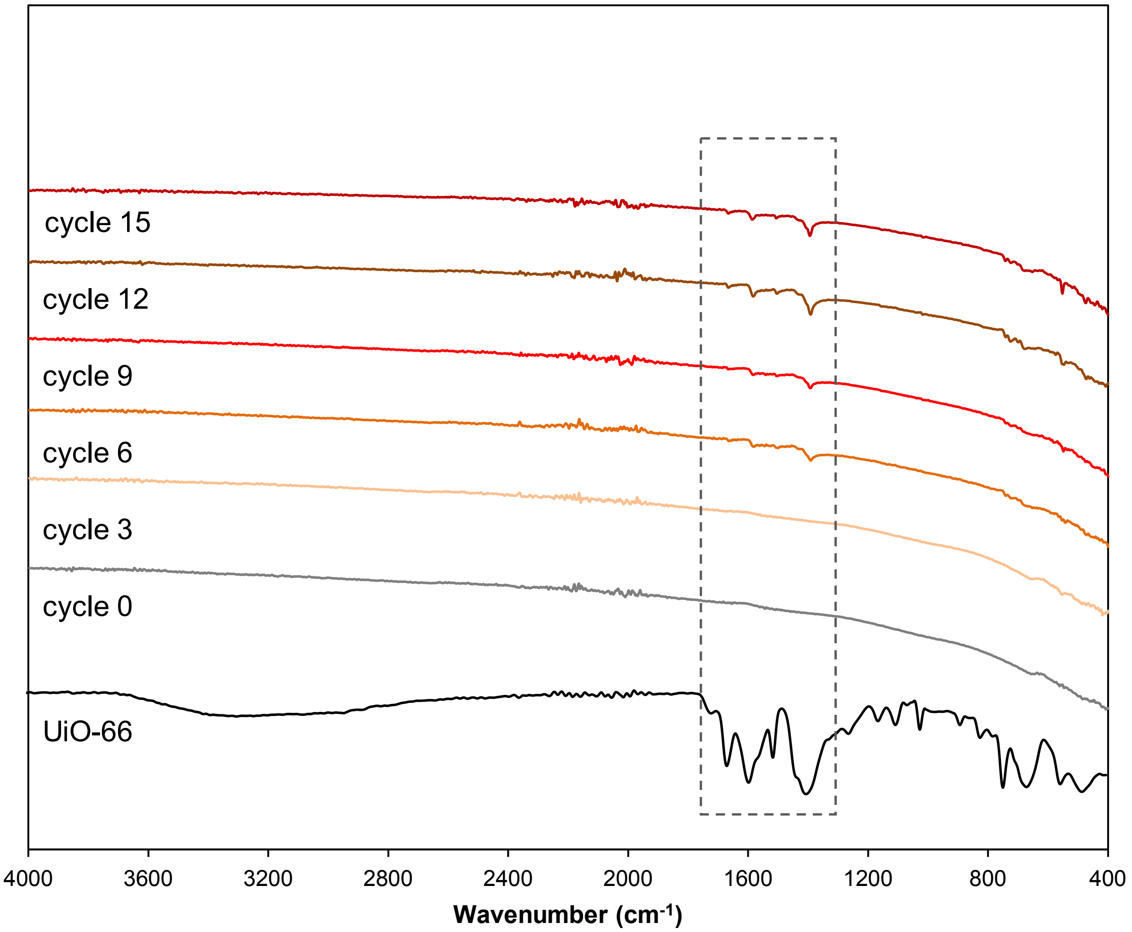

Figure S6. FTIR spectra for **UiO-66@AC(fabric)** after different synthetic cycles of 2h.

S3.3. Thermogravimetrical Analysis (TGA)

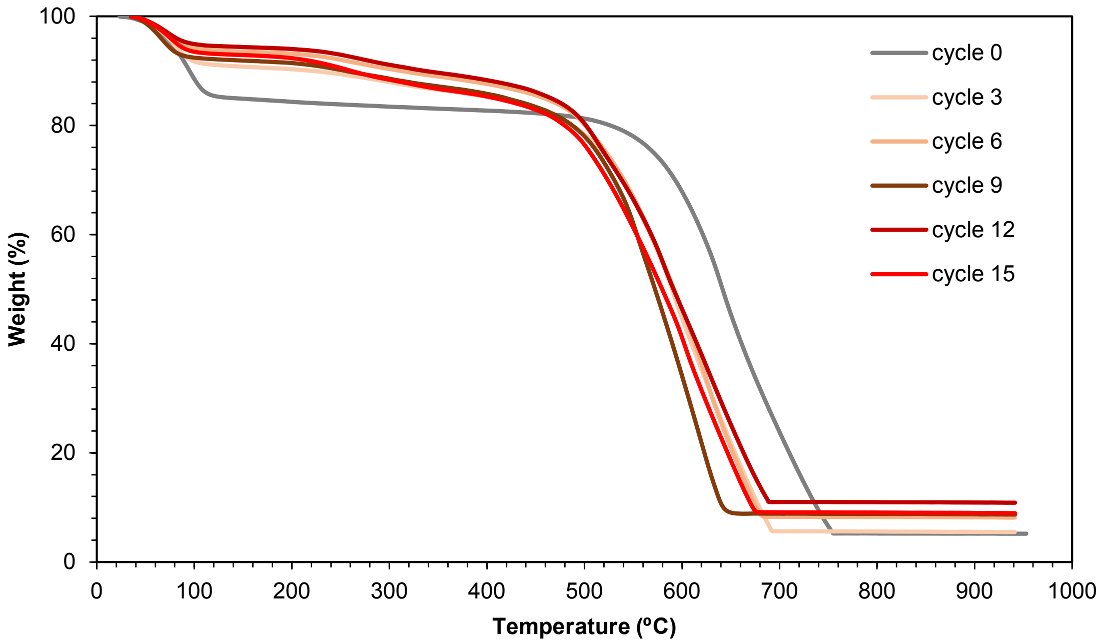

Figure S7. TGA analysis for **UiO-66@AC(fabric)** after different synthetic cycles of 2h.

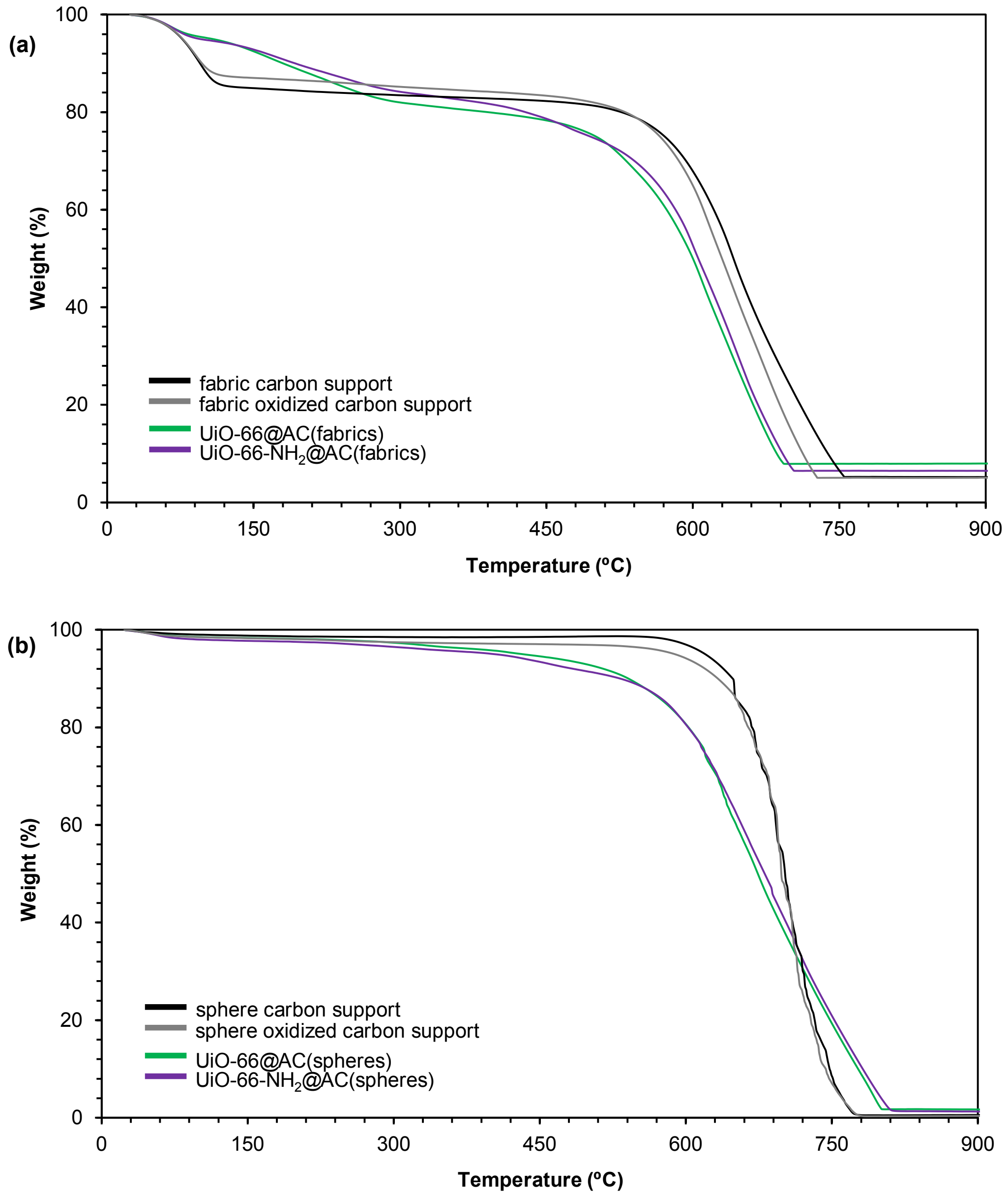

Figure S8. TGA analysis for (a) fabrics and (b) spheres prepared after 9 cycles at 130 °C (30 min total cycle).

S3.4. Scanning electron microscopy with energy dispersive X-ray spectroscopy (SEM-EDX)

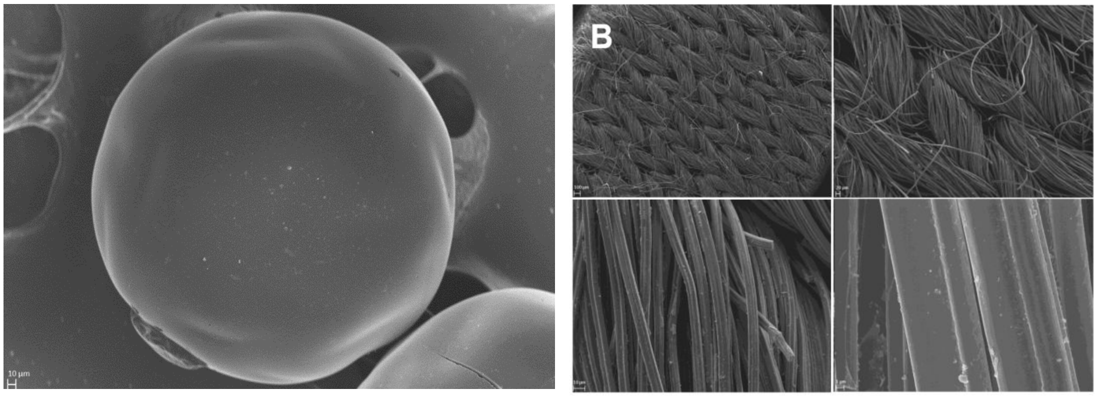

Figure S9. SEM images for pristine carbonaceous materials

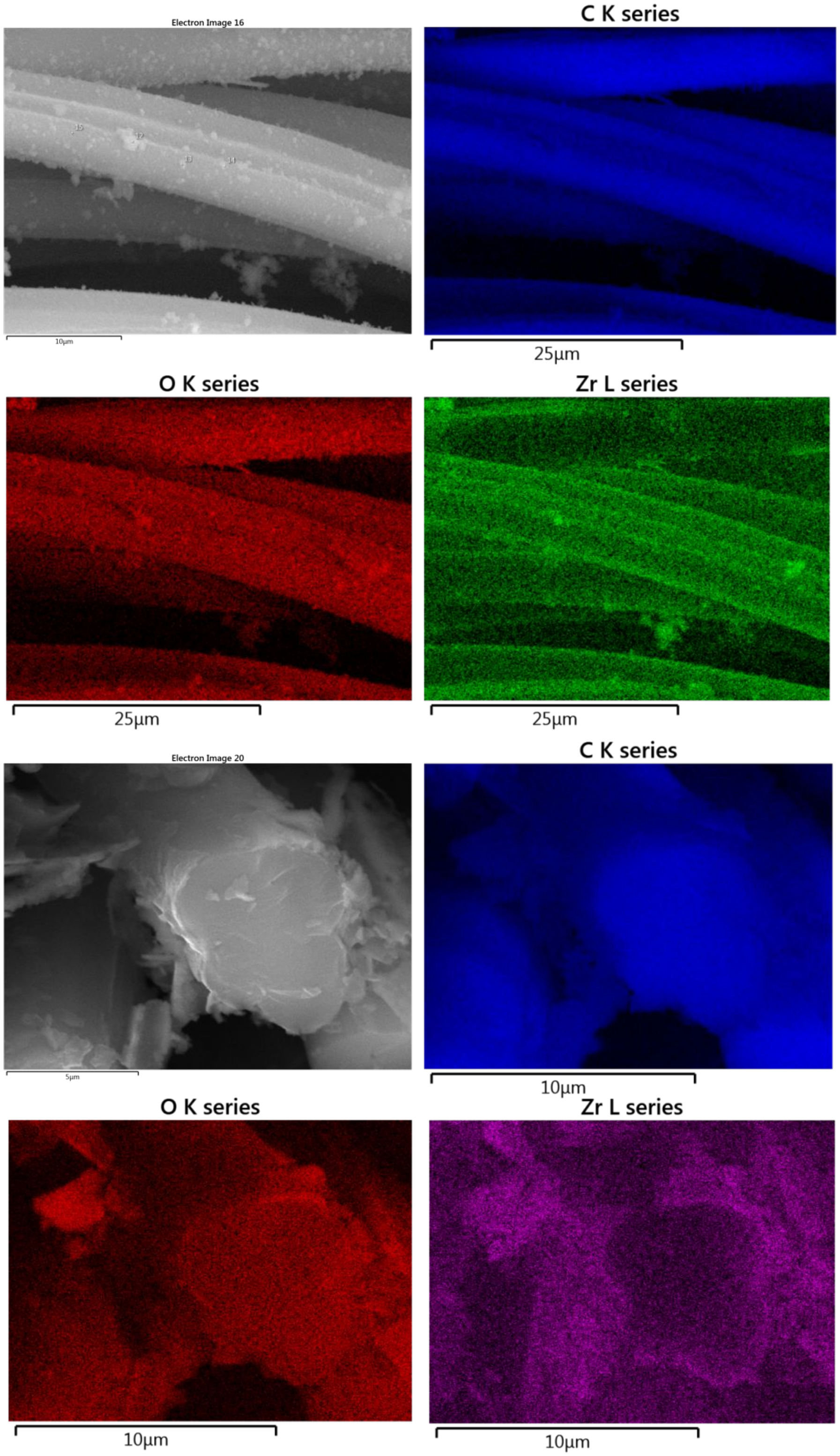
Electron Image 16
C K series
25µm
O K series
Zr L series
25µm
25µm
Electron Image 20
C K series
10µm
O K series
Zr L series
10µm
10µm

Figure S10. SEM images and EDX mapping for UiO-66@AC(fabrics) after 9 cycles (2h total cycle). Cross-sectional images and mapping are shown.

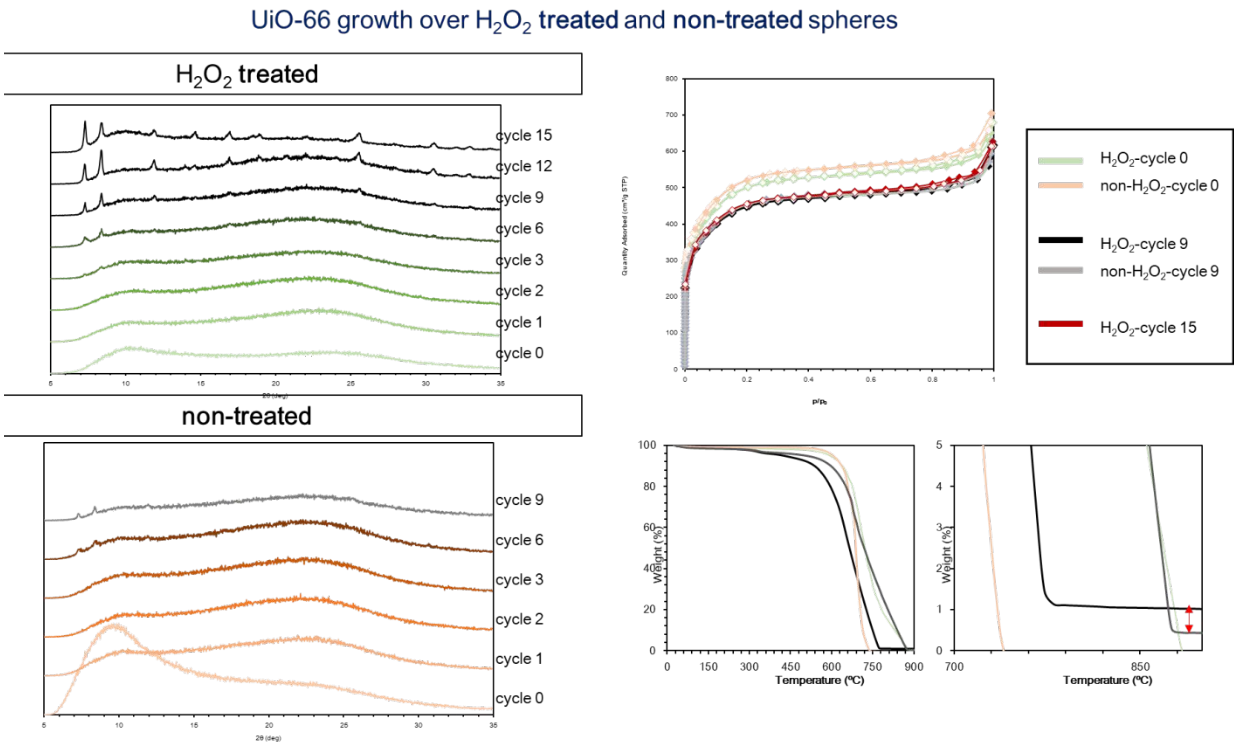

Figure S11. Impact of H_2O_2 treatment of carbon spheres on the layer by layer growth of UiO-66 as shown by XRPD (left), N_2 adsorption at 77 K and TGA.

S4. Catalytic tests

Heterogeneous catalytic degradation of DIFP nerve agent analogue.

We proceeded to the catalytic degradation of diisopropylfluorophosphate (DIFP) as a model of nerve CWA similarly to previous works by our group[3,4]. The degradation of DIFP was studied adding 40 mg of composites **UiO-66@AC(fabric)** and **UiO-66-NH$_2$@AC(fabric),** 10 µL of H_2O, and 1.25 µL of DIFP in a closed vial with a septum. The evolution of the concentration of DIFP was followed at 60°C. Due to the strong affinity of both DIFP and its degradation products to the activated carbon porous structure, a set of parallel experiments were carried out in order to evaluate the progress of the detoxification reaction. These experiments consisted in the extraction with 0.5 mL CH_2Cl_2 at a given time, namely, 1 min, 10 min, 1 h, 5 h, and 24 h. Afterwards, 1.25 µL of DMSO was added as an internal standard before measurement by means of Gas Chromatography employing an Agilent 30 m-column (0.53 mm internal diameter).

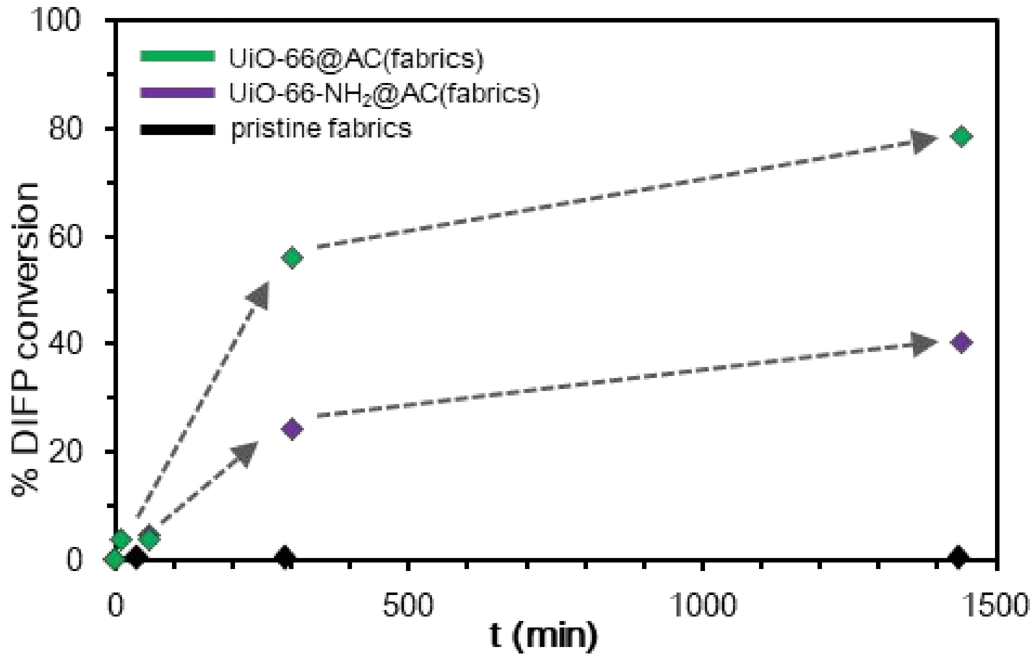

Figure S12. Blocking of the secondary emission of DIFP by MOF@AC fabric materials. Reaction conditions: 40 mg MOF@AC fabric catalyst, 1.25 µL DIFP, 10 µL H_2O, Aprox. MOF:DIFP ratio 1:5. 60°C. Extraction with CH_2Cl_2 (0.5 mL) at 1 min, 10 min, 1h, 5h and 24h.

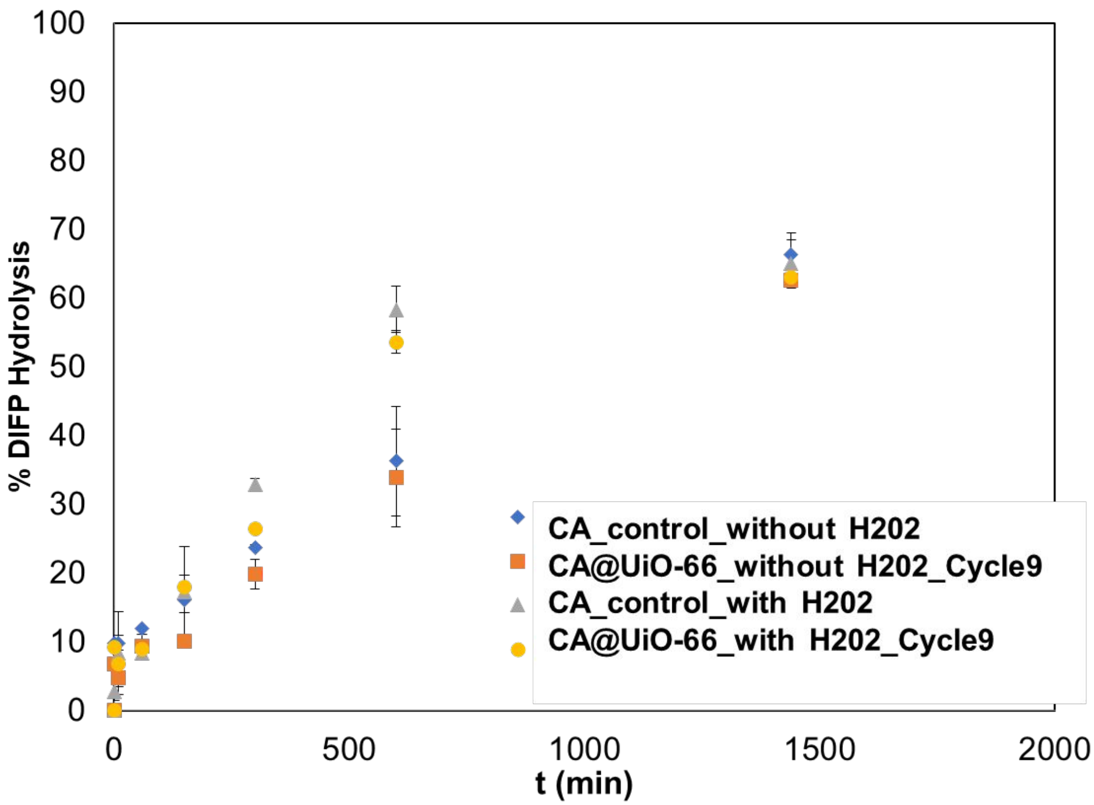

Figure S13. Study of the DIFP degradation by MOF@AC(sphere) materials. Reaction conditions: 40 mg MOF@AC(sphere) catalyst, 1.25 µL DIFP, 10 µL H_2O, Aprox. MOF:DIFP ratio 1:5. 60 °C. Extraction with CH_2Cl_2 (0.5 mL) at 1 min, 10 min, 1h, 5h and 24h.

S5. References

(1) DeStefano, M. R.; Islamoglu, T.; Garibay, S. J.; Hupp, J. T.; Farha, O. K. Room-Temperature Synthesis of UiO-66 and Thermal Modulation of Densities of Defect Sites. *Chem. Mater.* **2017**, *29* (3), 1357–1361. https://doi.org/10.1021/acs.chemmater.6b05115.

(2) Cavka, J. H.; Jakobsen, S.; Olsbye, U.; Guillou, N.; Lamberti, C.; Bordiga, S.; Lillerud, K. P. A New Zirconium Inorganic Building Brick Forming Metal Organic Frameworks with Exceptional Stability. *J. Am. Chem. Soc.* **2008**, *130* (42), 13850–13851. https://doi.org/10.1021/ja8057953.

(3) Gil-San-Millan, R.; López-Maya, E.; Platero-Prats, A. E.; Torres-Pérez, V.; Delgado, P.; Augustyniak, A. W.; Kim, M. K.; Lee, H. W.; Ryu, S. G.; Navarro, J. A. R. Magnesium Exchanged Zirconium Metal–Organic Frameworks with Improved Detoxification Properties of Nerve Agents. *J. Am. Chem. Soc.* **2019**, *141* (30), 11801–11805. https://doi.org/10.1021/jacs.9b05571.

(4) Gil-San-Millan, R.; López-Maya, E.; Hall, M.; Padial, N. M.; Peterson, G. W.; DeCoste, J. B.; Rodríguez-Albelo, L. M.; Oltra, J. E.; Barea, E.; Navarro, J. A. R. Chemical Warfare Agents Detoxification Properties of Zirconium Metal–Organic Frameworks by Synergistic Incorporation of Nucleophilic and Basic Sites. *ACS Appl. Mater. Interfaces* **2017**, *9* (28), 23967–23973. https://doi.org/10.1021/acsami.7b06341.
